# Prognostic Significance of Matrix Metalloproteinase-7 in Gastric Cancer Survival: A Meta-Analysis

**DOI:** 10.1371/journal.pone.0122316

**Published:** 2015-04-28

**Authors:** Saeed Soleyman-Jahi, Saharnaz Nedjat, Afshin Abdirad, Niloofar Hoorshad, Reza Heidari, Kazem Zendehdel

**Affiliations:** 1 Cancer Research Center, Cancer Institute of Iran, Tehran University of Medical Sciences, Tehran, Iran; 2 Epidemiology and Biostatistics Department, School of Public Health, Knowledge Utilization Research Centre (KURC), Tehran University of Medical Sciences, Tehran, Iran; 3 Medical school, Tehran University of Medical Sciences, Tehran, Iran; Heinrich-Heine-University and University Hospital Duesseldorf, GERMANY

## Abstract

The prognostic role of matrix metalloproteinase-7 in gastric cancer survival has been widely evaluated. However, the results are controversial. We aimed to set up a meta-analysis to reach a conclusion on the prognostic significance of metalloproteinase-7 in gastric cancer survival as well as its association with clinicopathological parameters. We searched popular databases from 1988 until October 2014 to gather eligible peer-reviewed papers addressing the prognostic effect of matrix metalloproteinase-7 in gastric cancer patients' survival. The CASP check list was used for quality appraisal. Pooled hazard ratio (HR) for survival and odds ratio (OR) for association with their 95% confidence interval (CI) were considered as summary measurements. Finally, 1208 gastric cancer patients from nine studies were included in the meta-analysis. Pooled HR estimate for survival was 2.01 (95% CI = 1.62 – 2.50, P < 0.001), which indicated a significant poor prognostic effect for matrix metalloproteinase-7. Sensitivity analysis detected no dominancy for any study. No publication bias was detected according to Egger’s and Begg’s tests. Clinicopathological assessment revealed that higher matrix metalloproteinase-7 expression is associated with deeper invasion (pooled OR = 3.20; 95% CI = 1.14 – 8.96; P = 0.026), higher TNM stage (pooled OR = 3.67; 95% CI = 2.281-5.99; P<0.001), lymph node metastasis (pooled OR = 2.84; 95% CI = 1.89 – 4.25; P<0.001), and distant metastasis (pooled OR = 3.68; 95% CI = 1.85 – 7.29; P<0.001), but not with histological grade. This meta-analysis indicated a significant poor prognostic effect of matrix metalloproteinase-7 in gastric cancer survival. Additionally it was associated with aggressive tumor phenotype.

## Introduction

Despite a recent decline in incidence, gastric cancer (GC) is still the second most frequent cause of cancer-related death worldwide [1]. GC patients are still diagnosed in a late stage and have a poor prognosis. Efficient diagnostic and prognostic modalities seem to be the missing parts in the approach for these patients [2]. Different prognosis observed for patients of the same clinical stage emphasizes the fact that the clinical stage cannot efficiently reflect the biological behavior of the tumor and new biological factors (e.g. biomarkers) are mandatory to complement clinical parameters for more precise decision-making [3].

Matrix metalloproteinase (MMPs) are among the cancer-related biomarkers that have recently attracted notable attention [4]. MMPs are a family of endogenous calcium- and zinc-dependent proteolytic enzymes that are capable of degrading most extracellular matrix (ECM) components, as well as regulating other enzymes, chemokines and even cell receptors. Twenty three types of MMPs have been described so far [5,6].

Many studies have investigated MMPs role in cancer progression. Systematic reviews and meta-analyses of these original reports conclude poor prognostic effects of MMP2 and MMP9 in stomach [7,8], breast [9,10], lung [11,12], colorectal [13,14] and ovarian [15] cancers; additionally they showed clinical significance of MMPs in bladder cancer [16] as well as prognostic effect of MMP7 in colorectal cancer [14]. This body of evidence strongly supports MMPs role in cancer progression.

MMP7, also called Matrilysin, is a distinct family member with proteolytic activity against a wide range of biomolecules including proteoglycans, laminin, fibronectin, casein and more importantly basement membrane collagen type IV [17,18]. It is recognized as pivotal in the MMP family since it activates other MMPs (i.e. MMP-2 and MMP-9) for ECM degradation [19] and possesses the highest activity in the MMP family [20]. Another specific characteristic of matrilysin in contrast to other MMPs is that it is mainly expressed by tumor cells and not by stromal cells [21–23]. Other than ECM degradation, MMP7 regulates many other cancer-supporting biochemical processes; it enhances cellular proliferation by increasing insulin-like growth factor and mature heparin-binding epidermal growth factor, cleaves cell to cell contact E-cadherin molecules, inhibits apoptosis in cancer cells [24,25] and induces angiogenesis [26]. Therefore, MMP7 could have a prominent prognostic role in tumors and merits comprehensive investigation. Many studies have assessed MMP7 role in cancer extension.

Elevated levels of MMP7 have been reported in many cancer types (gastric, esophageal, colorectal, pancreatic, prostate, head and neck, lung, hepatocellular and breast), as well as in cancer premalignant lesions (pancreas, stomach, colon, breast and prostate) [27]. In addition, MMP7 has been proposed as a prognostic factor in esophagus squamous cell cancer [23], non-small cell lung cancer [28] and in colorectal [29], breast [30], prostate [31] and urinary and bladder [32] cancers. MMP7 prognostic effect in GC has been widely investigated [3,27,33–40]. The original studies about the impact of MMP7 on patients' survival are not consistent [3,27,33–39]. A recent meta-analysis demonstrated that MMP7 level is significantly associated with clinicopathological parameters in GC [40]. However, this study did not include survival data such as hazard ratio or risk ratio. Association of biological markers with pathological parameters may or may not be linked to the patient outcome. Therefore, it is important to evaluate whether the observed association of the biomarkers with the baseline variables will affect the patients clinical outcome or not. Thus, we aimed to perform a meta-analysis to summarize existing survival data and reach a conclusion about the prognostic effect of MMP7 on the survival of GC patients.

## Materials and Methods

### Search strategy

A comprehensive search of electronic databases was completed from 1988 (when MMP7 was first introduced [41]) to October 29, 2014 in order to find clinical studies assessing the prognostic significance of MMP7 in GC. Databases searched included Medline, Embase, Web of Science, Google scholar, ProQuest (for dissertations) and Scopus. Key words were “matrix metalloproteinase 7”, “MMP-7” OR “matrilysin” AND ‘‘gastric” OR “stomach” AND “tumors”, ‘‘cancer”, ‘‘carcinoma”, ‘‘neoplasms” OR “CA” AND ‘‘survival,” ‘‘prognostic” or ‘‘prognosis.” The references cited in found full texts were scrutinized to find any additional studies not indexed in the databases that were searched. The language of the paper was not a matter of restriction for our search. We registered our review protocol in PROSPERO database (http://www.crd.york.ac.uk/PROSPERO) which can be accessed using registry number CRD42014013770 (S1 File).

### Study selection

Records in the primary search were assessed for relevance. The abstracts of relevant records were further screened to select appropriate items for full-text retrieval. Peer-reviewed and published studies addressing the association of MMP7 expression level (in blood or in resected gastric tumor specimen of histopathologically-confirmed gastric cancer patients) with patient survival were selected. A minimum of five years of follow-up was needed. Exclusion criteria were: *in vitro* and experimental studies, clinical cross-sectional studies, studies encompassing more than one type of cancer with no classified data, review articles, letters, editorials, conference abstracts and studies lacking the least requisite data to extract intended survival parameters for meta-analysis according to Palmar and Tierney [42,43]. When the results of the same cohort of patients were reported in more than one paper, the most informative study with the largest sample size was included. Eligible papers underwent quality appraisal and finally approved studies were included in the meta-analysis. We assumed no language restriction for study selection.

### Quality assessment

Two independent reviewers (SSJ and NH) scored the quality of selected papers using the critical appraisal skills program (CASP) cohort study quality assessment checklist (http://www.casp-uk.net/wpcontent/uploads/2011/11/CASP-Cohort-Study-Checklist-31.05.13). Then, they discussed their findings to reach a consensus on the final score of each paper. This checklist comprised 12 questions in three main parts (the validity of study, results and local implementation of results), and each appraised study received a score between 0 and 12. The quality scores achieved were used for subgroup analysis to check whether the quality of papers had an effect on the meta-analysis outcome or not.

### Data extraction

Already prepared data tables were used to extract the necessary information. Two reviewers (SSJ and NH) performed the extraction process independently and the following discrepancies were resolved upon consensus or following the third reviewer’s (KZ) decision. When necessary, we contacted the corresponding authors for the required data. The parameters extracted included: the first author of the study, year of publication, source country of the patients, ethnicities, sample size, specimen assessed for MMP7 expression level, method of quantitative assessment, scoring system for MMP7 measurements and cut-off values used, histological grade and stage of the patients, positive expression rate of MMP7, other clinicopathological parameters reported, follow-up duration and survival parameters (Hazard ratio (HR) and its 95% confidence interval (CI), overall survival (OS) rate, disease-specific survival (DSS) rate, peritoneal recurrence free survival (PRFS) rate, relative hazard (RH) and its 95% CI, Log-Rank test indices, and Kaplan-Meier survival curves). The intended main outcome measurement for meta-analysis was extracted using already described approaches [42–44]. The directly reported HR and its 95% CI were considered the most precise data. If not available, we tried to calculate it from Observed-Expected (O-E) event data in either group. Otherwise, we extracted the number of patients at risk, count of events and Log-Rank test indices to approximate HR and CI. Finally, if no informative numerical statistics were provided, survival curves were used. We exploited GetData Graph Digitizer software version 2.26.0.20 (http://getdata-graph-digitizer.com/) in order to handle the curves as precisely as possible. This was to tackle the inter-reader variability limitation of this approach [44]. Uniform censoring throughout the follow-up period was assumed and previously developed methods [42,43] were used to calculate the censored number of cases in each time interval of survival curve data extraction.

### Statistical methods

We used hazard ratios and the 95% CIs as the summary statistics for aggregated survival data, as already suggested [42] and the odds ratio (OR) and corresponding 95% CI to report aggregated association strength of MMP7 expression and other clinicopathological parameters. HR and OR above 1 were assumed to indicate poor prognosis and a positive association, respectively, provided that the 95% CIs did not overlap one.

Heterogeneity analysis was accomplished using both qualitative chi-square-based Q statistics and the quantitative metric I^2^ test (the number of studies analyzed does not affect the latter test) [45,46]. A *P* value < 0.05 for the Q statistics or I^2^ >50% indicated significant heterogeneity, necessitating a random-effect model for aggregated analysis. Otherwise, a fixed-effect model was used. I^2^ ≤ 50% would indicate a negligible quantitative degree of total variation among studies [45]. Sensitivity analysis was performed by the successive omission of individual studies to assess the integrity of summary results. Subgroup analysis based on intended parameters such as source country, measurement method and scoring system was performed to condition each parameter effect on summary results. Begg`s funnel plot and Egger`s test was exploited for potential publication bias assessment; an Egger`s test P value <0.10 would be interpreted as statistically significant [47].

We used Microsoft Excel 2013 for survival curves extracted data handling and Stata/SE version 11.1 software (Stata Corp LP, TX 77845, USA) for the rest of the analytical process.

## Results

### Literature information

**Fig 1:**
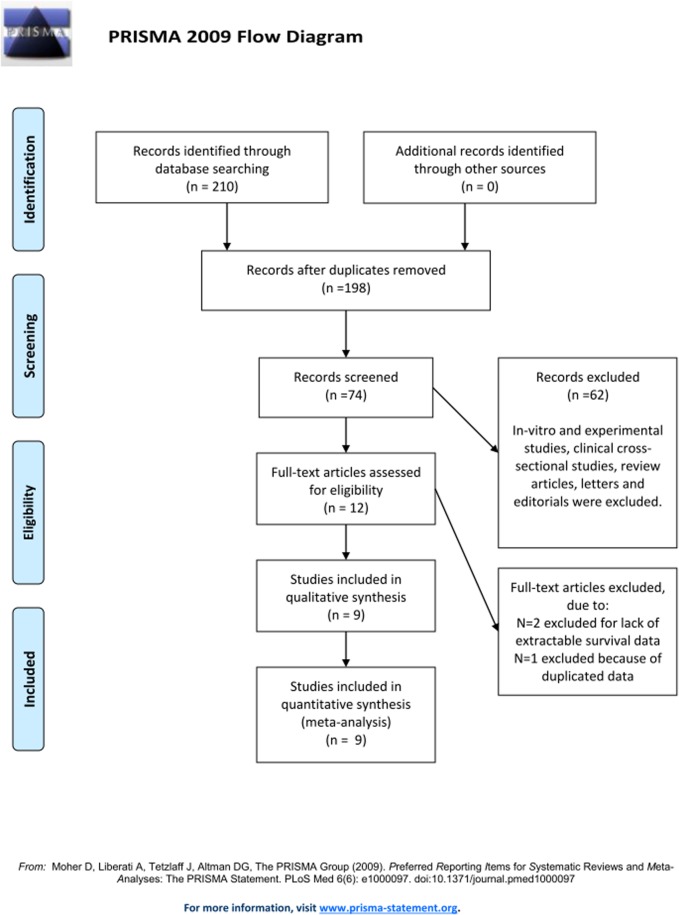
Flow diagram for study selection process. The figure demonstrates how finally included studies were selected from primary search records.

The initial search identified seventy four potentially relevant titles. By further reviewing the screening results, the reviewers determined twelve studies to be of acceptable relevance and format for the retrieval of full text. Among the selected papers, two [48,49] were excluded due to the lack of sufficient survival data and one [50] for data duplication; nine studies [3,27,33–39] met the eligibility criteria and requisite quality (Fig 1) and were included in analysis of the prognostic value of MMP7 in GC as well as its association with clinicopathological parameters.

### Study characteristics

Table 1 shows the main characteristics of the selected eligible studies. In the table, studies are categorized by source of the samples used for MMP7 assay. A total of 1208 patients (ranging 42–264 for each study) were included in the analysis. The mean age of the patients was 64.26 (individual study mean range of 53.5–67); also, 62.11% of them were male, 38.86% had TNM early stage/stage 1 or 2 and 35.09% had well differentiated grade GC. Six studies were of an Asian population [3,33,36–39] and the other three were from Finland [27], Brazil [35] and Spain [34]. IHC was used to assess MMP7 expression in five studies [3,27,33,35,38] and the other four exploited either a serum enzyme-linked immunosorbent assay (ELISA) [34,39] or real-time polymerase chain reaction (rt-PCR) [36,37]. Among the studies that used IHC, one study included invasive front of the tumor bulk in tissue sampling [38] and the others obtained specimens from random parts of the tumor [3,27,33,35]. Studies did not mention any control for IHC positivity. The MMP7 positive expression rate was 45–48.5% in four studies using IHC [3,27,33,35], and 66.7% in one using rt-PCR to detect MMP7 in different parts of gastric tumor specimens [36]; IHC-positive staining was up to 74.1% in the study assessing tumor invasive front [38]. The paper using rt-PCR to detect MMP7 mRNA in peritoneal lavage [37] reported a positive expression rate of 27%. The mean serum MMP7 concentration was reported to be 3.27 and 7.2 ng/ml in two studies using ELISA [34,39]. A 10–50% range of positive staining was used as the “high expression group” delineation cut-off point in IHC studies; it was 3.46 and 4.5 ng/ml of the serum MMP7 concentration in ELISA studies. Seven studies reported OS rate [3,27,33–36,39], one reported DSS rate [38] and the other reported PRFS rate [37]. Five out of nine included studies that finally concluded the poor prognostic effect of MMP7 in GC [27,33,36–38], while the other four did not reach such a conclusion [3,34,35,39]. A multivariate hazard ratio (HR) and its 95% confidence interval (CI) could be obtained for five studies (directly mentioned in the paper or sent by the corresponding author)[27,33,34,37,38]; just one of them reported direct univariate HR as well and we included multivariate HR for pooled analysis in this case [34]. For the rest of the studies [3,35,36,39], univariate HR was indirectly estimated from given Kaplan-Meier survival curves using previously described methods[42–44]. Studies achieved a score of 8–10 out of 12 in quality appraisal. Comprehensive confounding factor consideration and applicability of study results to the local population were items that most of studies did not meet.

**Table 1:** Main characteristics of nine included studies.

MMP7 source	First author (publish year)	Country	Sample size	Follow up (months)	+ MMP expression rate	Age (mean)	Male	I,II or early stage	Well diff. tumor	MMP7 assay method	Cut-off	HR and 95% CI	Extraction method	Analysis type	Study conclusion	Quality score*
Tissue	Liu X P (2002)	Japan	194	120	17.4–74.1%	67	58.9%	50.4%	24.7%	IHC	30%	2.67 (1.44–4.95)	direct	MV	poor	10
	Koskensalo S (2010)	Finland	264	56–250	48.5%	66	52%	34%	*NR*	IHC	50%	1.78 (1.14–2.79)	direct	MV	poor	10
	Ajisaka H (2004)	Japan	153	60	48%	*NR*	*NR*	49%	50%	IHC	50%	4.48 (2.26–8.90)	direct	MV	poor	10
	Fanelli M F (2012)	Brazil	137	60	45%	65	58.3%	47%	*NR*	IHC	*NR*	1.48 (0.70–3.15)	indirect	UV	not	9
	Sawada T (2014)	Japan	210	60	46.7%	*NR*	70%	50%	*NR*	IHC	10%	1.65 (1.07–2.53)	indirect	UV	*NR*	10
	LEE K H (2006)	Korea	42	72	66.7%	60	71%	23%	42%	rt-PCR	*NR*	2.59 (1.06–6.30)	indirect	UV	poor	8
Serum	Blanco-Calvo M (2014)	Spain	52	60	*NR*	66	81%	17.3%	40%	ELISA	3.5 ng/ml	1.02 (0.44–2.37)	direct	MV	not	9
	Yeh Y Ch (2010)	Taiwan	55	60	*NR*	53.5	58%	45%	10%	ELISA	4.5 ng/ml	2.10 (0.48–9.17)	indirect	UV	not	10
Peritoneal lavage	Li Z (2014)	China	116	60	27%	60.7	71%	0%	40%	rt-PCR	6.66 × 10^–3^	2.67 (1.11–6.46)	direct	MV	poor	10

*NR*: not reported in the study; *IHC*: immunohistochemistry; *ELISA*: enzyme-linked immunosorbent assay; *rt-PCR*: real-time polymerase chain reaction; *HR*: hazard ratio; *CI*: confidence interval; *UV*: univariate; *MV*: multivariate

* Quality scores are according to CASP tool for cohort studies assessment.

### Summary Hazard Ratio

**Fig 2:**
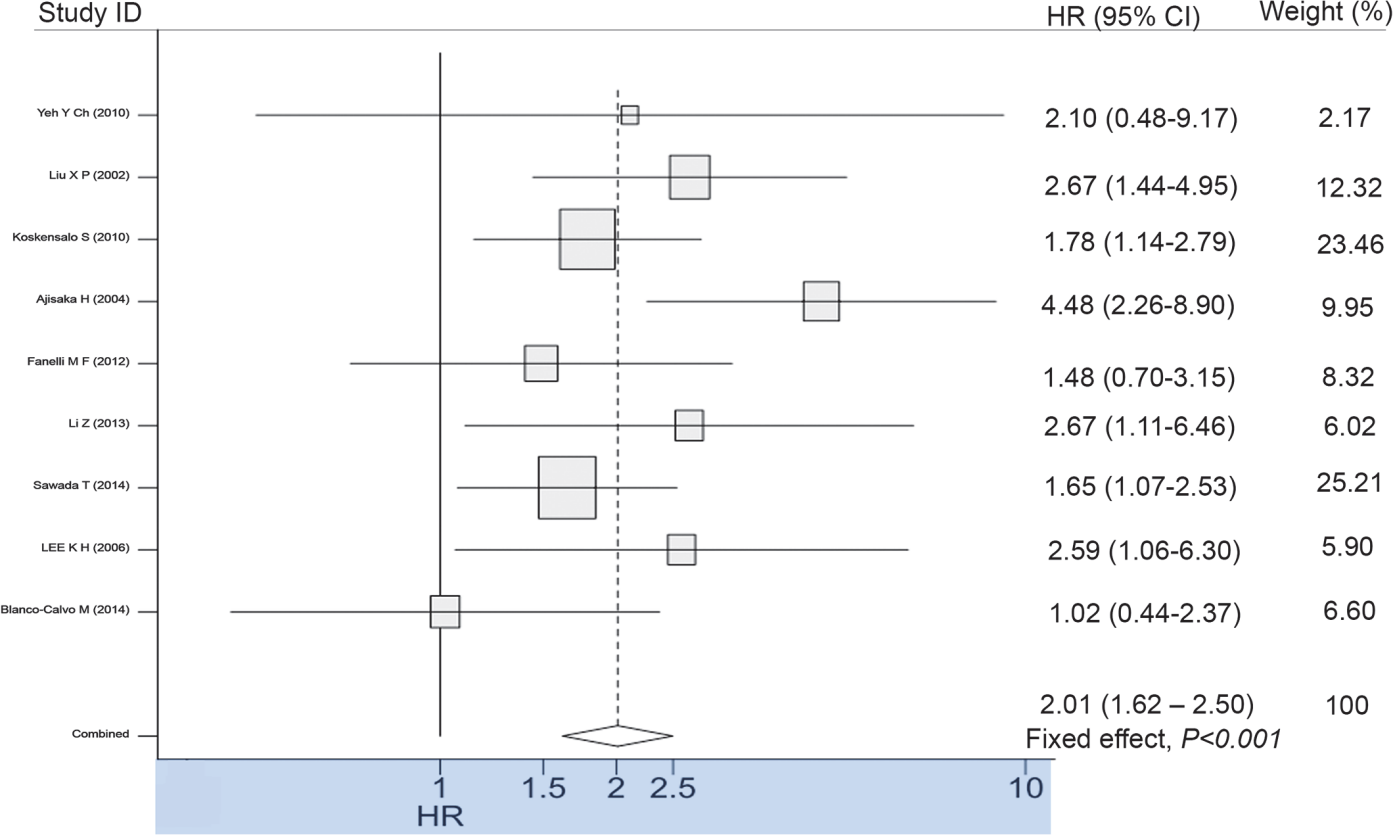
Forrest plot of overall hazard ratio estimate for MMP7 impact on GC survival. The middle point of the diamond represents the pooled HR and its left and right corners represent 95% CI. Horizontal lines belong to individual studies; the middle point and line length represent the corresponding study`s extracted HR and 95% CI. The area of box tagged with each line represents the individual study`s weight of contribution to the meta-analysis.

**Fig 3:**
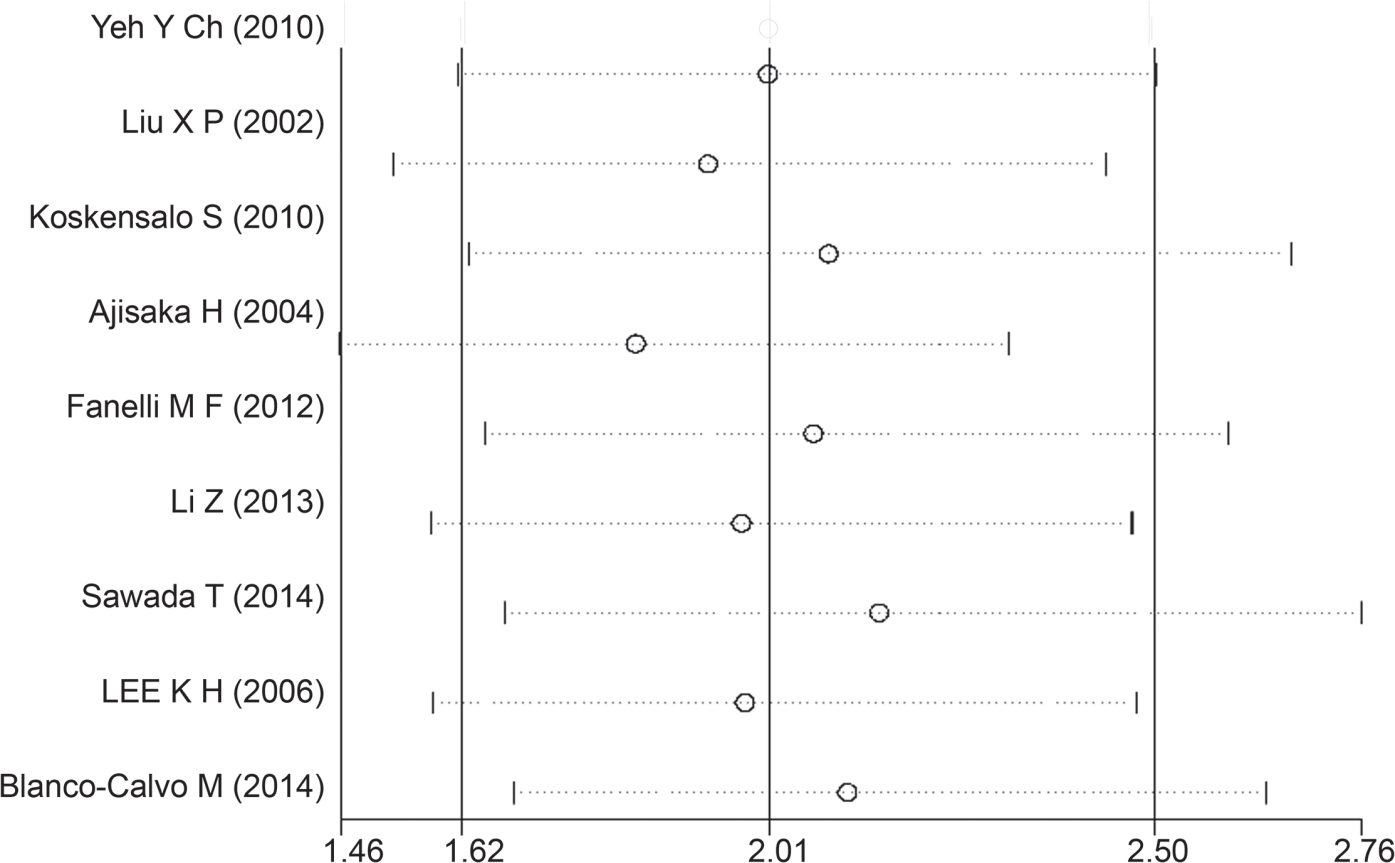
Diagram for each study effect on pooled HR of MMP7 impact on GC survival. The diagram illustrates sensitivity analysis results. Three bold vertical lines indicate pooled HR and it 95% CI when all studies included. Each dotted horizontal line belongs to a separate meta-analysis (with fixed effect model) when each respective study is omitted. The middle circle tagged represents corresponding pooled HR and two sides broken lines delineate its 95% CI.

Directly obtained multivariate HRs of five studies and indirectly estimated univariate HRs of four studies were included for aggregated survival analysis. Our meta-analysis indicated the significant poor prognostic effect of MMP7 in GC patients with insignificant heterogeneity (pooled HR = 2.01, 95% CI = 1.62–2.50, Z = 6.32, *P* < 0.001, Fixed effect; Q = 10.948 on 8 degrees of freedom, *P* = 0.205, estimate of between studies variance = 0.043). Pooled HR and its 95% CI forest plot are depicted in Fig 2. Sensitivity analysis was performed by the successive omission of each study from aggregated survival meta-analyses to examine the influence of each individual study on the pooled HR. Fig 3 shows that none of the estimated pooled HRs corresponding to the omission of each study was outside the 95% CI of the HR estimated from all studies in the overall, implying that no individual study was dominant in the pooled results.

**Fig 4:**
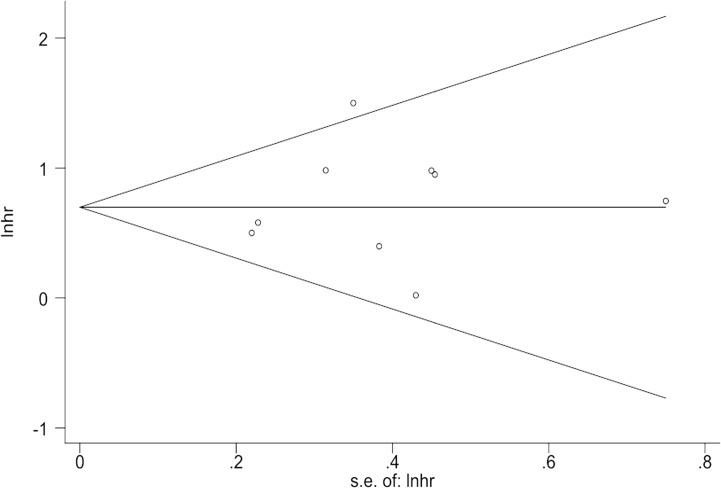
Begg`s funnel plot for publication bias in MMP7 impact on GC survival meta-analysis. Pseudo 95% Confidence limits are depicted and each hollow circle represents an individual study included in meta-analysis.

Neither Begg`s nor Egger`s tests showed significant publication bias for the studies included for summary analysis (Begg`s test Z = 0.21, *P* = 0.83; Egger`s test t = 0.21, *P* = 0.84). Begg`s publication bias funnel plot is illustrated in Fig 4. The figure does not show apparent asymmetry.

### Subgroup analysis

Pooled HR and heterogeneity tests results for overall data and data restricted to different subgroups are given in Table 2. Although none of the subgroups had significantly different pooled HR compared to overall or counterpart subgroups, the numerical values of pooled HRs restricted to Asian populations, tissue sample MMP7 assay and 50% IHC cut-off point studies were notably higher than the pooled HRs restricted to the non-Asian populations, serum sample MMP7 assay and <50% IHC cut-off point data, respectively. Only two studies used serum MMP7 and their pooled HR was not statistically significant. The pooled HR limited to studies reporting multivariate analysis was not notably different from the overall summary HR. Restricting analysis to studies with mainly high stage patients (when more than two-third of study cases were high stage) revealed a slightly lower pooled HR. The overall HR was comparable with HR restricted to studies reporting the OS rate. According to quality appraisal, the included studies had acceptable and comparable quality scores; thereafter, no categorization for quality score was considered to make a subgroup analysis.

**Table 2:** Subgroup meta-analysis results for MMP7 impact on Gastric Cancer survival.

Subgroups	Number of studies (number of patients)	HR (95% CI)	Z	P_Z_	P_Q_	Between studies variance estimate
All studies	9(1208)	2.01 (1.62–2.50)^a^	6.32	<0.001	0.20	0.04
Overall survival (OS) rate report	*7(878)*	*1*.*89 (1*.*48–2*.*40)* ^a^	*5*.*19*	*<0*.*001*	*0*.*15*	*0*.*07*
Large sample size	*5(943)*	*2*.*04 (1*.*60–2*.*60)* ^a^	*5*.*75*	*<0*.*001*	*0*.*10*	*0*.*08*
Multivariate analyses	*5(799)*	*2*.*23 (1*.*68–2*.*95)* ^a^	*5*.*53*	*<0*.*001*	*0*.*07*	*0*.*13*
Univariate analyses	*5(461)*	*1*.*89 (1*.*40–2*.*55)* ^a^	*4*.*16*	*<0*.*001*	*0*.*71*	*0*
Tissue sample	*6(985)*	*2*.*07 (1*.*64–2*.*62)* ^a^	*6*.*10*	*<0*.*001*	*0*.*16*	*0*.*05*
Serum sample	*2(107)*	*1*.*22 (0*.*59–2*.*54)* ^a^	*0*.*53*	*0*.*59*	*0*.*40*	*0*
IHC	*5(943)*	*2*.*04(1*.*60–2*.*60)* ^a^	*5*.*75*	*<0*.*001*	*0*.*101*	*0*.*08*
Other method	*4(265)*	*1*.*90 (1*.*18–3*.*05)* ^a^	*2*.*64*	*0*.*008*	*0*.*37*	*0*.*01*
Asian population	*6(790)*	*2*.*36 (1*.*79–3*.*10)* ^a^	*6*.*09*	*<0*.*001*	*0*.*28*	*0*.*03*
Other nationality	*3(418)*	*1*.*56 (1*.*01–2*.*21)* ^a^	*2*.*49*	*0*.*01*	*0*.*51*	*0*
Mainly high stage patients	*4(474)*	*1*.*82(1*.*31–2*.*55)* ^a^	*3*.*53*	*<0*.*001*	*0*.*33*	*0*.*01*
Comparable low and high stage patients	*5(734)*	*2*.*16 (1*.*62–2*.*87)* ^a^	*5*.*30*	*<0*.*001*	*0*.*12*	*0*.*10*
IHC cut off = 50%	*2(417)*	*2*.*72 (1*.*19–6*.*69)* ^b^	*2*.*18*	*0*.*03*	*0*.*03*	*0*.*34*
IHC cut off < 50%	*2(424)*	*1*.*93 (1*.*36–2*.*75)* ^a^	*3*.*65*	*<0*.*001*	*0*.*21*	*0*.*04*

*HR*: pooled hazard ratio; *CI*: confidence interval; *Z*: test value for fixed/random effect model; *P*_*Z*_: statistical *P value* for Z test; *P*_*Q*_: statistical *P value* for heterogeneity Q test.

^a^ Fixed effect model HR (95% CI)

^b^ random effect model HR (95% CI)

### Association of MMP7 with Clinicopathological Parameters

Table 3 shows the meta-analysis results. The elevated expression of MMP7 was significantly associated with more aggressive tumor characteristics such as deeper invasion (pooled OR = 3.20, P = 0.026; fixed effect), higher TNM stage (pooled OR = 3.67,P<0.001; fixed effect), lymph node metastasis (pooled OR = 2.84,P<0.001; random effect), lymphatic vessels infiltration (pooled OR = 2.39, P = 0.024; fixed effect), blood vessels infiltration (pooled OR = 2.03, P = 0.026; fixed effect) and distant metastasis (pooled OR = 3.68,P<0.001; fixed effect). No association was detected between MMP7 expression and tumor size, histologic grade, age and sex (Table 3).

**Table 3:** Meta-analysis of MMP7 overexpression association with clinicopathological parameters in included studies.

Parameter	Number of included cases	OR (95% CI)	Z	P_Z_	P_Q_	I^2^%
Age	319	1.70 (0.87–3.33)^b^	1.56	0.12	0.05	74.21
Sex	319	1.76 (0.88–3.50) ^a^	1.61	0.11	0.71	0
Tumor size	319	1.79 (0.84–3.82) ^a^	1.51	0.13	0.85	0
Depth	319	3.21 (1.15–8.97) ^a^	2.22	0.03	0.29	11.12
TNM stage	514	3.70 (2.28–5.99) ^a^	5.31	<0.001	0.07	58.11
Tumor differentiation	195	0.59 (0.31–1.01) ^a^	1.96	0.053	0.82	0
Lymph node metastasis	630	2.84 (1.89–4.25) ^b^	5.06	<0.001	0.01	65.21
Lymphatic vascular infiltration	195	2.39 (1.32–4.30) ^a^	2.89	0.004	0.14	68.12
Vascular invasion	195	2.03 (1.10–3.76) ^a^	2.26	0.02	0.08	67.11
Distant metastasis	319	3.68 (1.86–7.29) ^a^	3.74	<0.001	0.50	0

*OR*: pooled odds ratio; *CI*: confidence interval; *Z*: test value for fixed/random effect model; *P*_*Z*_: statistical *P value* for Z test; *P*_*Q*_: statistical *P value* for heterogeneity Q test. *I* ^*2*^ *%*: quantitative metric I^2^test.

^a^ Fixed effect model OR (95% CI)

^b^ random effect model OR (95% CI)

## Discussion

For the first time, to the best of our knowledge, this meta-analysis on 1208 patients' pooled data indicated the MMP7 expression level to be significantly associated with poor survival in GC patients; the high expression group pooled probability of death was estimated to be almost twice that of the low expression group. A fixed effect was used to report pooled results regarding the insignificant heterogeneity test. Sensitivity analysis showed that no individual study significantly dominated the pooled result. No publication bias was detected according to Begg's and Egger's tests. The clinicopathological data analysis in the same studies was meant to assess whether it is consistent with survival data; in support, it was shown that MMP7 level is significantly associated with aggressive tumor characteristics such as invasion depth, TNM stage, and distant metastasis.

By degrading ECM proteins and regulating the activity of other biomolecules in the body, MMPs mediate many processes such as cell migration, differentiation, proliferation, apoptosis, inflammatory reactions, and angiogenesis, both in physiological conditions (embryogenesis) and pathological disorders (cancer)[4,40]. The enzymes can affect pivotal steps of cancer biology such as growth, survival, angiogenesis and invasion [4]. MMP7 is a key member of this family that inherits the same properties as well as other specific features [19–21,26,51,52], which suggests that it is an affective biomolecule in tumorigenesis and cancer progression. Subsequent studies provided clinical evidence for these molecular findings [23,27–32]. Similar to other cancers, many authors aimed to assess the MMP7 prognostic role in GC survival. Some of them concluded that MMP7 is a poor prognostic factor for GC [27,33,36–38], while others[3,34,35,39] did not. Our meta-analysis concluded that MMP7 is a poor prognostic factor of GC survival.

A recent meta-analysis reported summary odds ratio data of MMP7 association with GC pathological indices [40]. The authors revealed that high MMP7 expression was associated with aggressive tumor phenotypes such as TNM stage, depth of tumor invasion, lymph node and distant metastasis. They detected no association between MMP7 and histological grade. Accordingly, in our included survival studies, we found that high MMP7 level is correlated with invasion depth, lymph node metastasis, distant metastasis and TNM staging. We did not find any significant association between MMP7 and grade either. The mentioned meta-analysis [40] did not include follow up survival data and did not address the inconsistency in results of studies that have investigated prognostic effect of MMP7 in gastric cancer.

Our subgroup analysis showed that pooled HR of Asian patients, tumor tissue MMP7 and 50% IHC cut-off point categories were numerically higher than the categories of non-Asian patients, serum MMP7 and <50% IHC cut-off point studies, respectively; however, they were all statistically comparable (as already shown by the insignificant heterogeneity test). Genetic background as well as environmental factors vary in different regions. This leads to tumor generation with different biological behavior [7]. In addition, tumor site differs between eastern and western countries, with consequent differences in tumor behavior and prognosis [53]. These could be reasons for the numerical differences detected between the pooled HR of Asian and non-Asian patients.

In patients with solid tumors, serum level of MMP7 does not seem to correlate strongly with tumor tissue level of MMP7. In breast, colorectal and ovarian cancers, no significant correlation was detected and just a weak correlation was reported in gastric cancer [54–57]. Accordingly, we found different results for prognostic effect of tissue and serum MMP7 level in gastric cancer. Although the summary estimates for association between tumor tissue MMP7 and prognosis of gastric cancer was significant, the pooled HR of two studies that used serum MMP7 was not statistically significant. Further studies are needed to assess association of serum MMP7 and the prognosis of gastric cancer.

Considering the higher values of MMP7-positive IHC staining as a cut-off to delineate the "high expression group" further discriminated between the prognosis of low and high expression groups and enhanced the specificity of MMP7 as a prognostic biomarker. Summary HR of studies with multivariate analysis data which encompasses about two-thirds of the included cases denoted a significant poor prognostic role of MMP7; this implies that the extracted summary prognostic effect of MMP7 in gastric cancer could be considered independent of other known prognostic factors (e.g. stage). Seven studies reported OS [3,27,33–36,39], one reported DSS [38] and the other reported PRFS [37]. Pooling data of three types of survival did not produce significant heterogeneity. In addition, pooled HR restricted to OS-reporting studies did not differ notably from overall HR.

The invasive front of tumor specimens showed a higher MMP7 expression rate compared to other parts of the tumor [38]. In addition, the type of antibody used and the degree of dilution could reveal different results [7]. Therefore, sampling site and IHC method (regarding type of antibody used and degree of dilution) should be standardized to address such measurement bias when introducing an IHC-based biomarker.

This study reached a homogeneous significant conclusion about the poor prognostic effect of MMP7 in GC patients' survival. This finding was supported by the association of MMP7 with aggressive tumor clinicopathological characteristics. Homogeneity further strengthened the meta-analysis and indicated that the analyzed data were similar enough to be pooled and that the summary results would be trustworthy. In general, HR > 2 is considered notably predictive [58]. Extracted prognostic effect of MMP7 in gastric cancer merits notice from two clinical aspects. It introduces MMP7 as a potential target for molecular anti-cancer therapy in gastric cancer. Current literature considers MMP7 a validated target for anticancer drugs [59]. Batimastat and Marimastat are broad spectrum MMP antagonists that target MMP7 along with some other MMPs [60,61]. There is experimental evidence for anti-proliferative and anti-metastatic effects of Batimastat [62]. Phase II and III clinical trials as well as observational studies demonstrate benefit of Marimastat administration in gastrointestinal malignancies [60,61,63,64]. Also an experimental study reports inhibitory effect of MMP7-specific antisense oligonucleotide on peritoneal dissemination in human GC [65]. In a certain type of cancer, a selection of MMPs with established poor prognostic effects could be a potentially good combination for targeted therapy.

In addition to be a target for GC therapy, MMP7 appears to be a good candidate for molecular staging of GC in the clinic to improve conventional clinical staging. It could help to characterize the patients more precisely and categorize them in appropriate therapeutic group. Further investigations are required to reach a consensus on appropriate method and cut-off values. In the biological milieu of tumor cells, the vast number of biomarkers were found to interact. Thereafter, an appropriately selected combination of biomarkers, instead of one item alone, could be considered a molecular signature of a tumor to complement the clinical staging.

Overall, the number of patients included in this meta-analysis as well as sample size in some of individual papers were small. This could limit the strength of our findings. However, pooled HR restricted to larger studies did not significantly differ from the overall pooled HR. Only five papers directly reported HRs; we extracted HR from survival curves in other studies. This method of data extraction is prone to bias [42]; we tried to limit this as much as possible (e.g. using a graphical curve reader software to read curves and choosing appropriate time intervals). Most studies did not consider a comprehensive profile of confounding factors to extract a very independent and pure prognostic effect of MMP7. Our search strategy targeted peer-reviewed published papers. Unpublished data and conference presentations were not included.

Future cohort studies with a larger number of patients would produce more robust results. In addition, well-designed multivariate survival analyses (such as multivariate Cox proportional hazard model) conditioning a comprehensive number of probable confounding variables are recommended to extract the independent pure prognostic effect of MMP7. A selection of appropriate molecular candidates along with MMP7 in combination with clinical data, could be assessed for outcome-predicting capability in GC. Such a study needs a very large sample size and requires collaboration of different groups and cancer centers.

For the first time, to the best of our knowledge, this meta-analysis on the statistically homogenous data from 1208 patients concluded that MMP7 is a poor prognostic factor for the survival of GC patients. Additionally, its overexpression was correlated with more advanced clinicopathological features. MMP7 alone, or more appropriately in combination with other biomarkers, could be considered a prognostic biomarker in the clinic to predict the outcome of GC patients, especially in Asian populations.

## Excluded Papers

1. Kim JH, Eom DY, Kim CW, Choi NK, Kwak JH, et al. (2011) Expression of E-cadherin, β-catenin, Cdx2 and MMP7 in pT2 and N1/N2 Gastric Cancer: Relationship with Tumor Recurrence within 2-Year Period. Journal of the Korean Surgical Society 80: 29–35 (Ref #35).
  Reason for exclusion: The paper does not present sufficient survival data (*Directly reported HR and its 95% CI*, *Observed-Expected (O-E) event data in either group*, *count of events and Log-Rank test indices or survival curves*) and we could not generate HR.
2. Kubben FJ, Sier CF, van Duijn W, Griffioen G, Hanemaaijer R, et al. (2006) Matrix metalloproteinase-2 is a consistent prognostic factor in gastric cancer. Br J Cancer 94: 1035–1040 (Ref #36).
  Reason for exclusion: The paper does not present sufficient survival data (*Directly reported HR and its 95% CI*, *Observed-Expected (O-E) event data in either group*, *count of events and Log-Rank test indices or survival curves*) and we could not generate HR.
3. Yonemura Y, Endou Y, Fujita H, Fushida S, Bandou E, et al. (2000) Role of MMP-7 in the formation of peritoneal dissemination in gastric cancer. Gastric Cancer 3: 63–70 (Ref # 40).
  Reason for exclusion: Gastric cancer patients included in this study are part of the patients included in the more recent paper "Ajisaka H, Yonemura Y, Miwa K (2004) Correlation of lymph node metastases and expression of matrix metalloproteinase-7 in patients with gastric cancer. Hepatogastroenterology 51: 900–905 (Ref # 33) ". The later paper used the same material with a larger sample size. Therefore, we decide to use the recent paper (Ref # 33) and excluded the first report (Ref # 40).

## Supporting Information

S1 PRISMA Checklist PRISMA checklist for systematic review studies. The file includes completed checklist for this study appraisal.
(DOC)

S1 File Registered review protocol. The file is a print of review protocol registered in PROSPERO review registration database.
(PDF)
