# Supplementary material for: Prognostic Significance of Matrix Metalloproteinase-7 in Gastric Cancer Survival: A Meta-Analysis
Source: PLoS One. 2015 Apr 28;10(4):e0122316. doi: 10.1371/journal.pone.0122316 (PMC4412628; doi:10.1371/journal.pone.0122316)
Supplement: S1 File — The file is a print of review protocol registered in PROSPERO review registration database. (PDF) [file pone.0122316.s002.pdf]

PROSPERO International prospective register of systematic reviews

Review title and timescale

1 Review title

Give the working title of the review. This must be in English. Ideally it should state succinctly the interventions or exposures being reviewed and the associated health or social problem being addressed in the review.

Measuring matrix metalloproteinase 7 expression level as prognostic factor for gastric cancer patients survival

2 Original language title

For reviews in languages other than English, this field should be used to enter the title in the language of the review. This will be displayed together with the English language title.

3 Anticipated or actual start date

Give the date when the systematic review commenced, or is expected to commence.

14/05/2014

4 Anticipated completion date

Give the date by which the review is expected to be completed.

05/10/2014

5 Stage of review at time of this submission

Indicate the stage of progress of the review by ticking the relevant boxes. Reviews that have progressed beyond the point of completing data extraction at the time of initial registration are not eligible for inclusion in PROSPERO. This field should be updated when any amendments are made to a published record.

The review has not yet started ×

Review stage	Started	Completed
Preliminary searches	Yes	Yes
Piloting of the study selection process	Yes	Yes
Formal screening of search results against eligibility criteria	Yes	Yes
Data extraction	Yes	Yes
Risk of bias (quality) assessment	Yes	Yes
Data analysis	Yes	Yes

Provide any other relevant information about the stage of the review here.

Review team details

6 Named contact

The named contact acts as the guarantor for the accuracy of the information presented in the register record.

Dr Soleyman-Jahi

7 Named contact email

Enter the electronic mail address of the named contact.

ty.jahi@yahoo.com

8 Named contact address

Enter the full postal address for the named contact.

Tehran University of Medical sciences, Poorsina street, Enghelab avenue, Tehran. Iran

9 Named contact phone number

Enter the telephone number for the named contact, including international dialing code.

0989143919441

10 Organisational affiliation of the review

Full title of the organisational affiliations for this review, and website address if available. This field may be completed as 'None' if the review is not affiliated to any organisation.

Cancer Research Center, Cancer Institute of Iran, Tehran University of Medical Sciences

Website address:

11 Review team members and their organisational affiliations

Give the title, first name and last name of all members of the team working directly on the review. Give the organisational affiliations of each member of the review team.

Title	First name	Last name	Affiliation
Dr	saeed	Soleyman-Jahi	Cancer Research Center, Cancer Institute of Iran, Tehran University of Medical Sciences, Tehran, Iran
Dr	Saharnaz	Nedjat	Epidemiology and Biostatistics Department, School of Public Health, Knowledge, Utilization Research Centre (KURC), Tehran University of Medical Sciences, Tehran, Iran
Dr	Afshin	Abdirad	
Dr	Niloofar	Hoorshad	
Dr	Reza	Heidari	
Dr	Kazem	Zendehdel	

12 Funding sources/sponsors

Give details of the individuals, organizations, groups or other legal entities who take responsibility for initiating, managing, sponsoring and/or financing the review. Any unique identification numbers assigned to the review by the individuals or bodies listed should be included.

Grant number 93-01-51-25150 from Tehran University of Medical Sciences (http://research.tums.ac.ir) supports this project.

13 Conflicts of interest

List any conditions that could lead to actual or perceived undue influence on judgements concerning the main topic investigated in the review.

Are there any actual or potential conflicts of interest?

None known

14 Collaborators

Give the name, affiliation and role of any individuals or organisations who are working on the review but who are not listed as review team members.

Title	First name	Last name	Organisation details
Dr	Medi	Eslani	Department of Ophthalmology and Visual Sciences, University of Illinois at Chicago

Review methods

15 Review question(s)

State the question(s) to be addressed / review objectives. Please complete a separate box for each question.

What is the prognostic significance of tissue or serum matrix metalloproteinase 7 expression level in gastric cancer patients survival?

16 Searches

Give details of the sources to be searched, and any restrictions (e.g. language or publication period). The full search strategy is not required, but may be supplied as a link or attachment.

Comprehensive search of intended electronic databases was accomplished up to September 10, 2014 to find clinical studies assessing prognostic significance of MMP7 in GC. Searched databases included PubMed, EMBASE, Web of Science, Google Scholar, ProQuest (for dissertations) and Scopus. No restriction were considered.

17 URL to search strategy

If you have one, give the link to your search strategy here. Alternatively you can e-mail this to PROSPERO and we will store and link to it.

I give permission for this file to be made publicly available

Yes

18 Condition or domain being studied

Give a short description of the disease, condition or healthcare domain being studied. This could include health and wellbeing outcomes.

Gastric Cancer

19 Participants/population

Give summary criteria for the participants or populations being studied by the review. The preferred format includes details of both inclusion and exclusion criteria.

Histopathologically confirmed gastric cancer patients

20 Intervention(s), exposure(s)

Give full and clear descriptions of the nature of the interventions or the exposures to be reviewed

Tissue or plasma matrix metalloproteinase seven expression level (measured with IHC,ELISA or rt-PCR)

21 Comparator(s)/control

Where relevant, give details of the alternatives against which the main subject/topic of the review will be compared (e.g. another intervention or a non-exposed control group).

Other clinical staging systems (e.g. TNM staging system).

22 Types of study to be included initially

Give details of the study designs to be included in the review. If there are no restrictions on the types of study design eligible for inclusion, this should be stated.

Prospective survival studies with at least five years of follow up.

23 Context

Give summary details of the setting and other relevant characteristics which help define the inclusion or exclusion criteria.

In-vitro and experimental studies, clinical cross-sectional studies, studies encompassing more than one type of cancer with no classified data, review articles, letters, editorials, conference abstracts were excluded.

24 Primary outcome(s)

Give the most important outcomes.

Survival (measured with hazard ratio or risk ratio indices)

Give information on timing and effect measures, as appropriate.

25 Secondary outcomes

List any additional outcomes that will be addressed. If there are no secondary outcomes enter None.

Association with clinicopathological indices (stage, invasion depth, ...)

Give information on timing and effect measures, as appropriate.

26 Data extraction, (selection and coding)

Give the procedure for selecting studies for the review and extracting data, including the number of researchers involved and how discrepancies will be resolved. List the data to be extracted.

Two reviewers (SSJ and NH) accomplished the extraction process independently and following discrepancies were resolved upon consensus or the third reviewer (KZ) decision. When needed, we contacted corresponding authors for required data. The parameters extracted included: the first author of the study, year of publication, source country of the patients, ethnics, sample size, specimen assessed for MMP7 expression level, method of quantitative assessment, scoring system of MMP7 measurement and used cut-off values, histological grade and stage of the patients, positive expression rate of MMP7, other clinicopathological parameters reported, follow up duration and survival parameters (Hazard ratio (HR) and its 95% confidence interval (CI), overall survival(OS) rate, disease specific survival (DSS) rate , peritoneal recurrence free survival (PRFS) rate, relative hazard (RH) and its 95% CI, Log-Rank test indices, Kaplan-Meier survival curves).

27 Risk of bias (quality) assessment

State whether and how risk of bias will be assessed, how the quality of individual studies will be assessed, and whether and how this will influence the planned synthesis.

Two independent reviewers (SSJ and NH) will score the quality of selected papers using critical appraisal skills

program (CASP) cohort study quality assessment checklist (http://www.casp-uk.net/wp content /uploads /2011 /11 /CASP-Cohort-Study-Checklist-31.05.13). Then they will discuss to reach a census on final score of each paper. This checklist consists of 12 questions in three main parts (the validity of study, results and local implementation of results) and each appraised study would get a score between 0 and 12. The quality scores achieved, if so varied, would be used for subgroup analysis to check if the quality of papers impresses meta-analyzed outcome or not.

28 Strategy for data synthesis

Give the planned general approach to be used, for example whether the data to be used will be aggregate or at the level of individual participants, and whether a quantitative or narrative (descriptive) synthesis is planned. Where appropriate a brief outline of analytic approach should be given.

We will use hazard ratio and its 95% CI as the summary statistics for aggregated survival data as already suggested [Parmar MK, Torri V, Stewart L (1998) Extracting summary statistics to perform meta-analyses of the published literature for survival endpoints. Stat Med 17:2815-2834.] and odds ratio (OR)and corresponding 95% CI to report aggregated association strength of MMP7 expression and other clinicopathological parameters.

29 Analysis of subgroups or subsets

Give any planned exploration of subgroups or subsets within the review. ‘None planned’ is a valid response if no subgroup analyses are planned.

Sensitivity analysis will be done by successive omission of individual studies to assess summary results integrity. Subgroup analysis based on intended parameters such as source country, measurement method and scoring system will be performed to condition each parameter effect on summary results.

Review general information

30 Type of review

Select the type of review from the drop down list.

Prognostic

31 Language

Select the language(s) in which the review is being written and will be made available, from the drop down list. Use the control key to select more than one language.

English

Will a summary/abstract be made available in English?

Yes

32 Country

Select the country in which the review is being carried out from the drop down list. For multi-national collaborations select all the countries involved. Use the control key to select more than one country.

Iran

33 Other registration details

List places where the systematic review title or protocol is registered (such as with he Campbell Collaboration, or The Joanna Briggs Institute). The name of the organisation and any unique identification number assigned to the review by that organization should be included.

34 Reference and/or URL for published protocol

Give the citation for the published protocol, if there is one.

Give the link to the published protocol, if there is one. This may be to an external site or to a protocol deposited with CRD in pdf format.

I give permission for this file to be made publicly available

Yes

35 Dissemination plans

Give brief details of plans for communicating essential messages from the review to the appropriate audiences.

Do you intend to publish the review on completion?

Yes

36 Keywords

Give words or phrases that best describe the review. (One word per box, create a new box for each term)

Matrix Metalloproteinase-7

Gastric cancer

survival

systematic review;

meta-analysis

prognosis

37 Details of any existing review of the same topic by the same authors

Give details of earlier versions of the systematic review if an update of an existing review is being registered, including full bibliographic reference if possible.

38 Current review status

Review status should be updated when the review is completed and when it is published.

Completed but not published

10/01/2015

39 Any additional information

Provide any further information the review team consider relevant to the registration of the review.

40 Details of final report/publication(s)

This field should be left empty until details of the completed review are available.

Give the full citation for the final report or publication of the systematic review.

Give the URL where available.
